# SLAM-MS: Mutation scanning of stem-loop amplicons with TaqMan probes by quantitative DNA melting analysis

**DOI:** 10.1038/s41598-020-62173-x

**Published:** 2020-03-25

**Authors:** V. N. Kondratova, I. V. Botezatu, V. P. Shelepov, A. V. Lichtenstein

**Affiliations:** N.N. Blokhin Russian Cancer Research Center, Ministry of Health of Russia, 24 Kashirskoye Shosse, Moscow, 115478 Russia

**Keywords:** Cancer, Genetics, Biomarkers, Medical research, Oncology

## Abstract

DNA Melting Analysis (DMA) with a TaqMan probe covering the mutation “hot spot” is a simple, sensitive, and “closed tube” method of mutation detection. However, DMA requires asymmetric PCR to produce single-stranded amplicons capable of interacting with TaqMan probes. This makes quantitative analysis impossible owing to low amplification efficiency. Moreover, bi-strand mutation detection necessitates two independent PCRs. The SLAM-MS (**S**tem-**L**oop **AM**plicon **M**utation **S**canning) assay, in which symmetric PCR is performed using primers with 5'-universal primer sequence (UPS), has been developed to detect *KRAS* mutations. Some of the resulting amplicons, sense and antisense, adopt single-stranded stem-loop conformation and become unable to renature, but able to hybridize with TaqMan probes. Hybrids of stem-loops and complementary TaqMan probes are suitable for melting analysis and simultaneous bi-strand mutation scanning. In addition, the areas under the melting peaks are determined by the PeakFit software, a non-linear iterative curve fitting program, to evaluate the wild-type/mutant allele ratio. Thus, the SLAM-MS assay permits quantification of both the number of copies of the target sequence and the percentage of mutant alleles. For mutant enrichment, the SLAM-MS assay uses TaqMan probes as PCR blocking agents allowing an ~10 times higher mutation detection sensitivity than High Resolution Melting (HRM) assay.

## Introduction

In basic cancer research, priority is given to large-scale (genome- and exome-wide) next generation sequencing (NGS) methods, which give a panoramic picture of defects in the cancer genome. However, for clinical diagnostics, a “targeted approach” is more often required, i.e. screening of previously known clinically important mutations, in particular in the *KRAS, NRAS, BRAF, EGFR*, and *PIK3CA* genes^1–7^. In this case, the priorities are the feasibility of the method in a clinical laboratory, and its simplicity, performance, and sensitivity.

These requirements are largely met by asymmetric PCR with a TaqMan probe covering the mutation “hot spot” and subsequent DMA (DNA Melting Analysis), which is one of the most simple, fast, and least costly methods to detect DNA mutations. Moreover, it is implemented in the “closed tube format” that avoids any additional manipulations and eliminates sample cross-contamination^8,9^. The short length of the TaqMan probes (20–30 nucleotides) facilitates strong mismatch-induced changes in melting curves and clear discrimination of wild-type and mutant melt peaks. Computation of the areas under the peaks using specialized software permits quantification of the ratio of wild and mutant alleles^9^. Furthermore, under special (suboptimal) PCR conditions, the TaqMan probes serve as blocking agents contributing to the enriched synthesis of mutant alleles^10^.

An inherent drawback of this method is the asymmetric mode of PCR necessary for accumulation of single-stranded amplicons (“targets” for TaqMan probes). This leads to a number of limitations: (*i*) a decrease in the amplification efficiency, and the inability to estimate the copy number of the target sequence; (*ii*) the need for two independent PCRs for bi-strand mutation scanning; (*iii*) increased costs of time and labour. A simple solution to the problem is the use of combined primers, consisting of 5'-universal and 3'-specific sequences, in symmetric PCR. This leads to the formation of single-stranded stem-loop amplicons along with the typical double-stranded amplicons. The stem-loop conformation precludes single strands from interactions with each other owing to topologic constraints, but creates a constant environment for hybridization with TaqMan probes. This is the basis for the quantitative SLAM-MS (stem-loop amplicon mutation scanning) assay that includes a number of technical novelties: (*i*) multifaceted use of TaqMan probes as real time indicators of symmetric amplification, blocking agents, and hybridization probes for DNA melting analysis; (*ii*) one-test bi-strand mutation scanning; (*iii*) quantitation of DNA melting analysis. In conclusion, the bi-strand mutant-enriched SLAM-MS seems very promising as a simple, moderately sensitive, cost-effective, and quantitative method of the “first line” diagnostics.

To develop the SLAM-MS assay, the *KRAS* oncogene was used as a prototype. This is one of the most clinically significant genes, exhibiting mutations in ~40% of colon cancer cases^11^.

## Material and Methods

### DNA samples

Samples of blood and tumour tissue (colon cancer) were obtained at the N.N. Blokhin Russian Cancer Research Center clinic. All patients consented to the use of their tissue samples. Formalin-Fixed Paraffin-Embedded (FFPE) or fresh-frozen primary tissues were used for detection of *KRAS* mutations (codons 12 and 13). DNA from the human colorectal carcinoma cell line SW480 (ATCC CCL-228, Manassas, VA, USA) with a homozygous mutation in the *KRAS* codon 12 (GGT/GTT)^12^ was used for serial dilutions of mutant DNA with wild-type DNA. DNA from blood cells of healthy donors, cultured cells, and tumour tissues was isolated using a proteinase K and phenol-chloroform deproteinization method. DNA was extracted from FFPE tissues using the QIAamp DNA FFPE tissue kit (QIAGEN, Valencia, CA, USA) as per the manufacturer’s protocol. DNA concentrations were determined spectrophotometrically (Nano-Drop 1000, Thermo Fisher Scientific, Wilmington, DE, USA).

### PCR design

**Figure 1:**
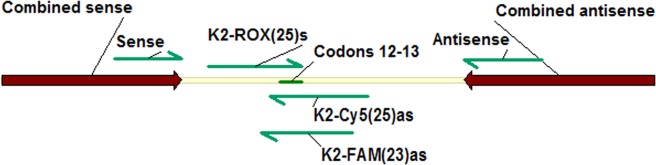
Scheme of the amplicons K2(114) and K2(174). The locations of the primers (standard and combined with the UPS sequence), as well as the TaqMan probes and the mutation “hot spot” (codons 12/13) are shown by arrows.

Thermodynamic calculations of Tm for primers and probes were performed using the MeltCalc program^13^. Folding of single-stranded amplicons was determined using the Mfold web server^14^. Primers for the *KRAS* sequence (GenBank Accession number NG_007524.1) were designed using the Vector NTI Advance 10 program (Invitrogen Corp., Carlsbad, CA, USA). The sequences of primers and probes are presented in Table 1. The scheme of the *KRAS* amplicons is shown in Fig. 1. The TaqMan probes are “shifted” relative to each other to prevent their mutually absorbing hybridization. Amplicons of 114 and 174 bp were synthesized using the standard and combined primers, respectively. The combined primers contain a universal GC-enriched Universal Primer Sequence (UPS) at the 5′-end^15^.

**Table 1:** Primers and TaqMan probes.

Standard primers [amplicon K2(114)]	
Forward	5'-gcctgctgaaaatgactg
Reverse	5'-ttggatcatattcgtccacaa
**Combined primers [amplicon K2(174)]**	
Forward	5'-***GCGGGCGTACTAGCGTACCGCTAGCGACGG***gcctgctgaaaatgactg
Reverse	5'-***GCGGGCGTACTAGCGTACCGCTAGCGACGG***ttggatcatattcgtccacaa
**TaqMan probes**	
K2-ROX(25)s	5'-ROX-acttgtggtagttggagctggtggc-BHQ2
K2-Cy5(25)as	5'-Cy5-aaggcactcttgcctacgccaccag-BHQ2
K2-FAM(23)as	5'-FAM-cactcttgcctacgccaccagct-RTQ1

The Universal Primer Sequence (UPS) is shown in italics and bold; specific sequences are indicated by lowercase letters; the TaqMan probe name indicates the amplicon, fluorophore, oligonucleotide length and direction (sense and antisense probes are designated by signs ‘s’ and ‘as’, respectively).

### SLAM-MS

PCR was performed in 25-μl reactions in 96-well plates on a CFX96 instrument (Bio-Rad Laboratories, Hercules, CA). The incubation mixture contained 67 mM Tris-HCl, pH 8.8; 16.6 mM (NH4)_2_SO_4_; 0.01% Tween 20; 2.5 mM MgCl_2_; 0.25 mM each of deoxynucleoside triphosphates; 0.2 μM forward and reverse combined primers; 0.2 μM TaqMan probes (sense and antisense, either alone or together); 1 unit Hot-rescue Taq polymerase (Syntol, Russia); 5 μl of DNA solution (10–30 ng) in water. The PCR conditions included an initial denaturation of 5 min at 95 °C; followed by 55 cycles of 25 s at 95 °C, 30 s at 56 °C, and 30 s at 72 °C, with fluorescence acquisition at 72 °C. Subsequently, the PCR products were denatured for 3 min at 95 °C, then cooled in one of three modes: average cooling rate (directly in the instrument), slowly (at room temperature), quickly (by immersing the plate in a water–ethanol mixture pre-cooled to −20 °C). DNA was melted, raising the temperature from 50 °C to 85 °C (increments of 0.4 °C, dwell time of 6 s, rate of heating of 3.3 °C/s). Data were analysed using the Bio-Rad CFX Manager program (version 1.6). Intercalating dyes (such as SYBR Green and EvaGreen) were not added to the incubation medium.

### Mutant-enriched SLAM-MS

PCR conditions were as described above except that after primary DNA denaturation (5 min at 95 °C), a two-stage PCR was performed: 50 short cycles (15 s at 95 °C, 1 s at 50 °C) preceded the standard 35 cycles (25 s at 95 °C, 30 s at 56 °C, 30 s at 72 °C). The DNA melting conditions remained the same.

### Quantitation of DNA melting analysis

**Figure 2:**
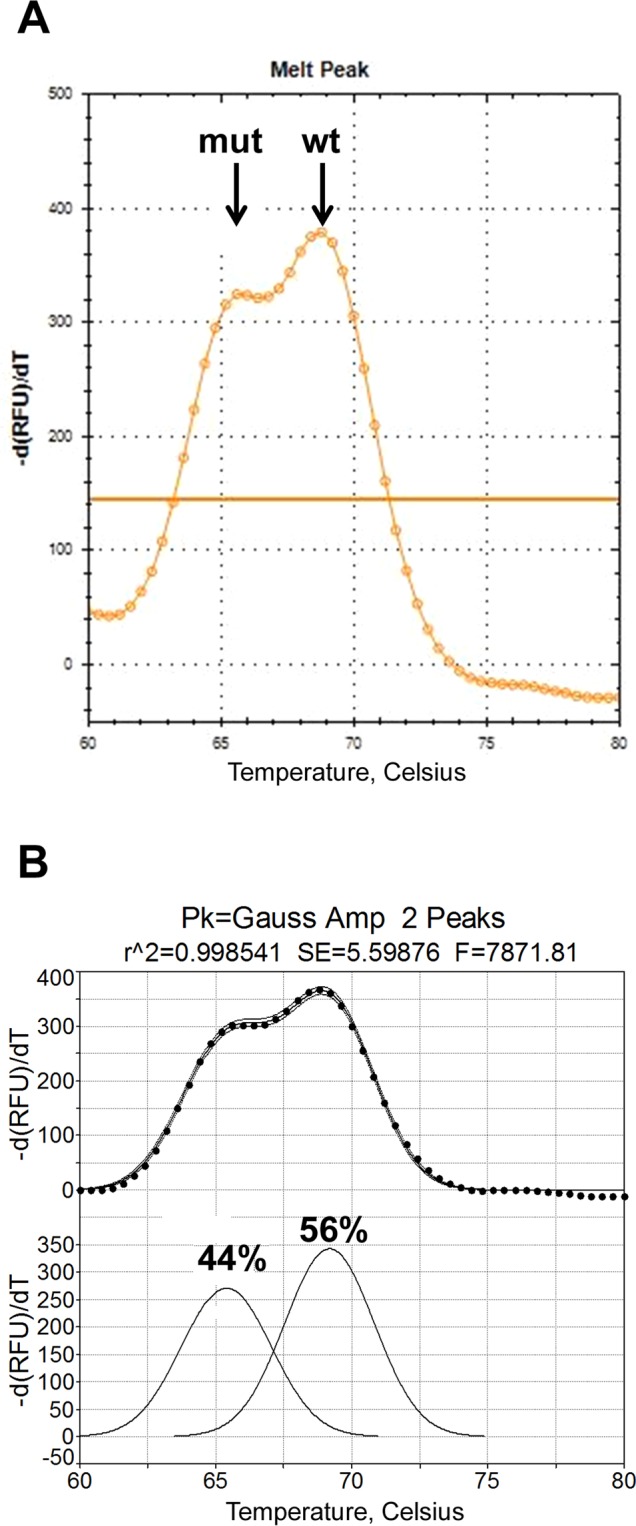
DMA quantitation. (**A**) *KRAS* GGT12GTT mutation from colorectal cancer was assayed by DMA with K2-ROX(25)s probe. (**B**) The melt peaks were subjected to the signal processing procedure using the PeakFit software. Upper panel - the fitted melt peak curve after background subtraction within 99% confidence interval; lower panel - separated melt peaks and their percentages.

Quantitation of DNA melt peaks was carried out as described earlier^9^. We assume that the number ratio of mutant and wild-type alleles has a linear relationship with the area ratio of respective melting peaks. The latter were calculated using PeakFit (v. 4.12.00; SeaSolve Software Inc., San Jose, CA, USA), a nonlinear peak separation software for spectroscopy and chromatography analyses. An iterative least-squares curve fitting procedure was employed to measure the areas under the curves of separate and overlapping Gaussian peaks after background subtraction. The goodness of curve fitting criteria, such as the *R*^2^ coefficient of determination, the standard error, 95–99% confidence interval, and the *F*-statistics for the fit are presented (Fig. 2). The correlation between the mutation fraction measured by the Peak Fit software and the actual mutation fraction obtained by serial dilutions of SW480 DNA with wild-type DNA was determined using GraphPad Prism 8.2.0 (GraphPad Software, San Diego, CA, USA).

### High resolution melting analysis

PCR was performed in 25-μl reactions in 96-well plates on a CFX96 instrument (Bio-Rad Laboratories, Hercules, CA). The incubation mixture contained 67 mm Tris-HCl, pH 8.8; 16.6 mM (NH4)_2_SO_4_; 0.01% Tween 20; 2.5 mM MgCl_2_; 0.2 μM forward and reverse K2(114) primers; 1.25 μl of intercalating dye 20 x EvaGreen (Dialat, Moscow, Russia); 0.25 mM each of deoxynucleoside triphosphates; 1 unit Hot-rescue Taq polymerase (Syntol, Moscow, Russia); 5 μl of DNA solution (10 ng) in water. The PCR conditions included an initial denaturation of 5 min at 95 °C; followed by 40 cycles of 25 s at 95 °C, 30 s at 56 °C, and 30 s at 72 °C (fluorescence acquisition at 72 °C) and a final extension at 72 °C for 5 min. Subsequently, the PCR products were denatured for 3 min at 95 °C, then cooled 3 min at 50 °C. DNA was melted, raising the temperature from 50 °C to 95 °C (increments of 0.2 °C, dwell time of 10 s, rate of heating of 3.3 °C/s). Data were analysed using the Bio-Rad Precision Melt Analysis Software 1 (v. 4.0.52.0602).

### Gel electrophoresis

DNA was separated by electrophoresis in 7% standard polyacrylamide gel, prepared in 0.5 x Tris-acetate buffer, pH 8.0, 6 h at 100 V (5 V/cm) and 4 °C. The gel was stained with EtBr (0.5 μg/ml).

### Single strand conformation polymorphism (SSCP)

Electrophoresis in a neutral gel with a high degree of crosslinking and under high voltage was carried out in a 12% polyacrylamide gel (acrylamide/bisacrylamide ratio 1:50), with 0.5 x Tris-acetate buffer, pH 8.0, for 2.5 h at 400 V (20 V/cm) and 4 °C^16,17^. The gel was stained with SYBR Gold fluorescent dye (dilution 1:10000).

### Sanger sequencing

*KRAS* amplicons were sequenced bi-directionally at the Syntol sequencing facility (Moscow) using the Sanger sequencing method.

## Results

Experimental and clinical studies typically require evaluation of the copy number of a target gene and the presence of polymorphisms, particularly mutations. Quantitative real time PCR with TaqMan probes is often used to evaluate copy number, and post-PCR DMA to detect polymorphisms^8,9,18^. A disadvantage of this approach is that the quantitative real time PCR is symmetric, while DMA with TaqMan probes requires an asymmetric PCR; therefore, two independent specific reactions are necessary. In this study, we combined symmetric PCR and DMA in one assay by using primers with 5'-universal primer sequence. As a result, parts of the sense and antisense single-stranded amplicons adopt the stem-loop conformation^15^ and are able to hybridize with complementary TaqMan probes.

### Products of PCR amplification

**Figure 3:**
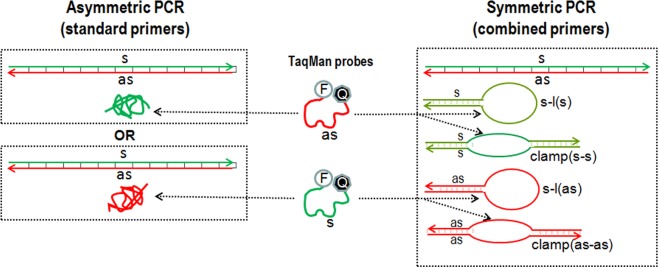
Possible structures resulting from asymmetric PCR with standard primers and symmetric PCR with combined primers. Designations: s - sense, as - antisense, s-l - stem-loops, F - fluorophore, Q - quencher. Interactions of TaqMan probes with single-stranded amplicons and stem-loops are indicated by arrows.

The comparison of possible structures arising during asymmetric PCR with standard primers and symmetric PCR with combined primers is presented in Fig. 3. In both cases, a significant portion of PCR products are ordinary double-stranded amplicons (rods) that cannot interact with TaqMan probes. In addition, asymmetric PCR produces a certain number of single-stranded amplicons (random coils) of one particular type, either sense or antisense, capable of hybridizing with a complementary probe.

**Figure 4:**
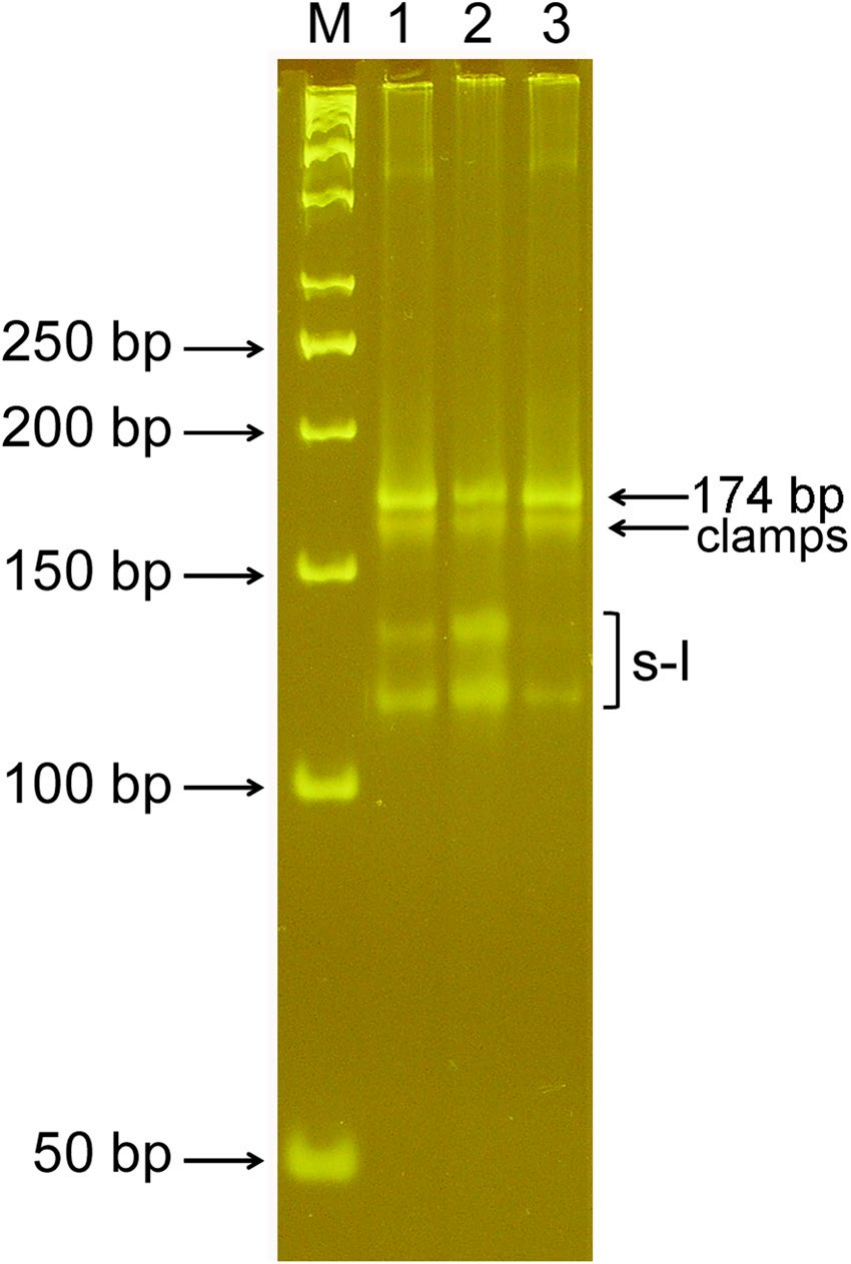
Electrophoresis of PCR products. PCR products synthesized by symmetric PCR with combined primers were separated in a 7% polyacrylamide gel after various modes of renaturation. Lanes: 1 - instrument cooling; 2 - quick cooling; 3 - slow cooling. Designations: M - molecular weight markers. s-l - stem-loops.

In comparison, symmetric PCR with combined primers should generate a more diverse range of products: (*i*) double-stranded rods, (*ii*) single-stranded stem-loops (sense and antisense), (*iii*) partially complementary “clamps” (sense-sense and antisense-antisense). The stem-loops and clamps have single-stranded regions probably capable of hybridizing with complementary probes. These assumptions were tested by electrophoretic analysis of products synthesized by symmetric PCR of this type (Fig. 4).

In addition to the expected 174 bp amplicon band, three further bands were observed. These presumably represent, in order of increasing migration rate, a single band of clamps and two bands of stem-loops. One can suggest that the ratios between the competitive conformations may change depending on renaturation conditions. Indeed, perfect complementary rods are the most thermodynamically favourable and, therefore, should be dominant. But, the bimolecular reaction of hybridization is concentration- and time-dependent, while the formation of stem-loops is an intramolecular, concentration-independent reaction that proceeds very quickly. So, the faster the cooling of amplicons after denaturation, the higher the expected proportion of stem-loop amplicons. The clamps are less thermodynamically advantageous than rods and slower than stem-loops. So, their proportion should be negligible. These assumptions are supported experimentally: fast cooling leads to an increase in intensity of the stem-loop bands (compare tracks 1, 2, and 3 in Fig. 4) and enlarged DNA melt peaks (Supplementary Fig. S1). Thus, it seems reasonable to perform the renaturation step at the maximal cooling rate for the instrument. In most cases, the instrumental cooling is enough to produce an adequate signal, but sometimes accelerated (outside the instrument) cooling of the plate and repeated melting may be recommended to enhance the DMA signal.

**Figure 5:**
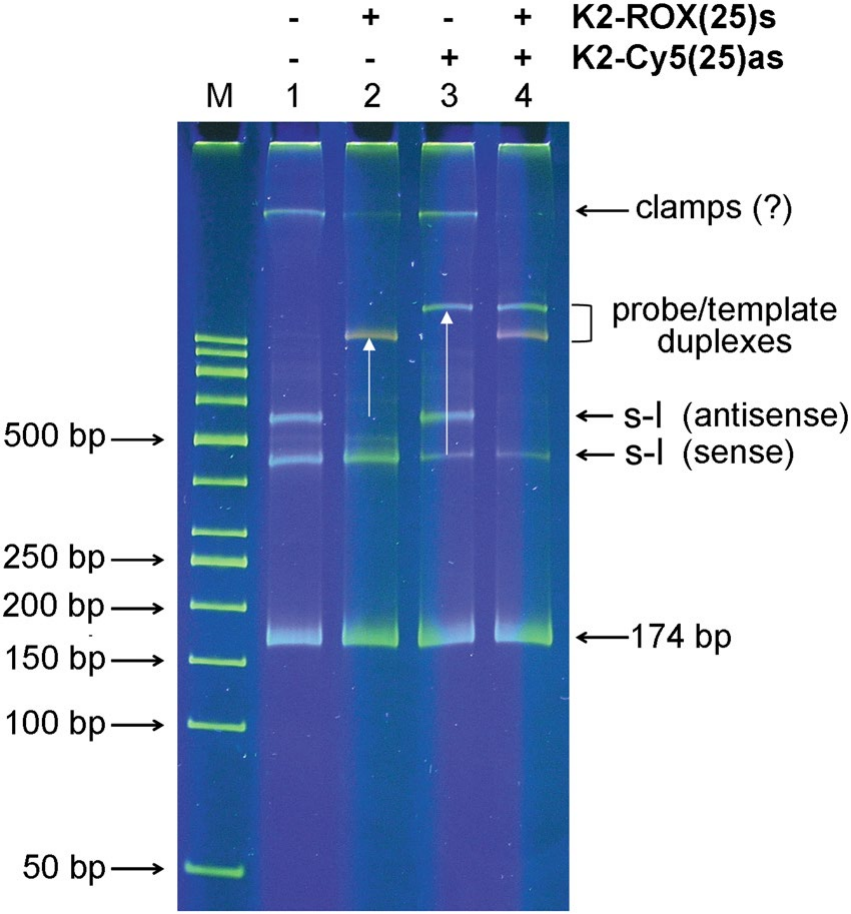
Band-shift experiments. Products of symmetric PCR incubated with TaqMan probes were separated by SSCP electrophoresis. Designations: M - molecular weight markers, s-l - stem-loops. Band shifts are indicated by arrows.

In subsequent band-shift experiments, PCR products were analysed using the SSCP method to evaluate the possibility of interactions between stem-loops and TaqMan probes. During SSCP electrophoresis designed for the analysis of conformational polymorphisms^16,17^, single-stranded coils move slower than linear rods and are resolved better than in standard electrophoresis. The band shifts arising when the probes K2-ROX(25)s and/or K2-Cy5(25)as are added to the amplification products indicate that stem-loop/probe interactions are possible (Fig. 5). It is also evident that K2-ROX(25)s hybridizes more efficiently than K2-Cy5(25)as, which can be explained by various steric effects of their specific fluorophores.

### Mutant-enriched SLAM-MS with quantitative DNA melting analysis

**Figure 6:**
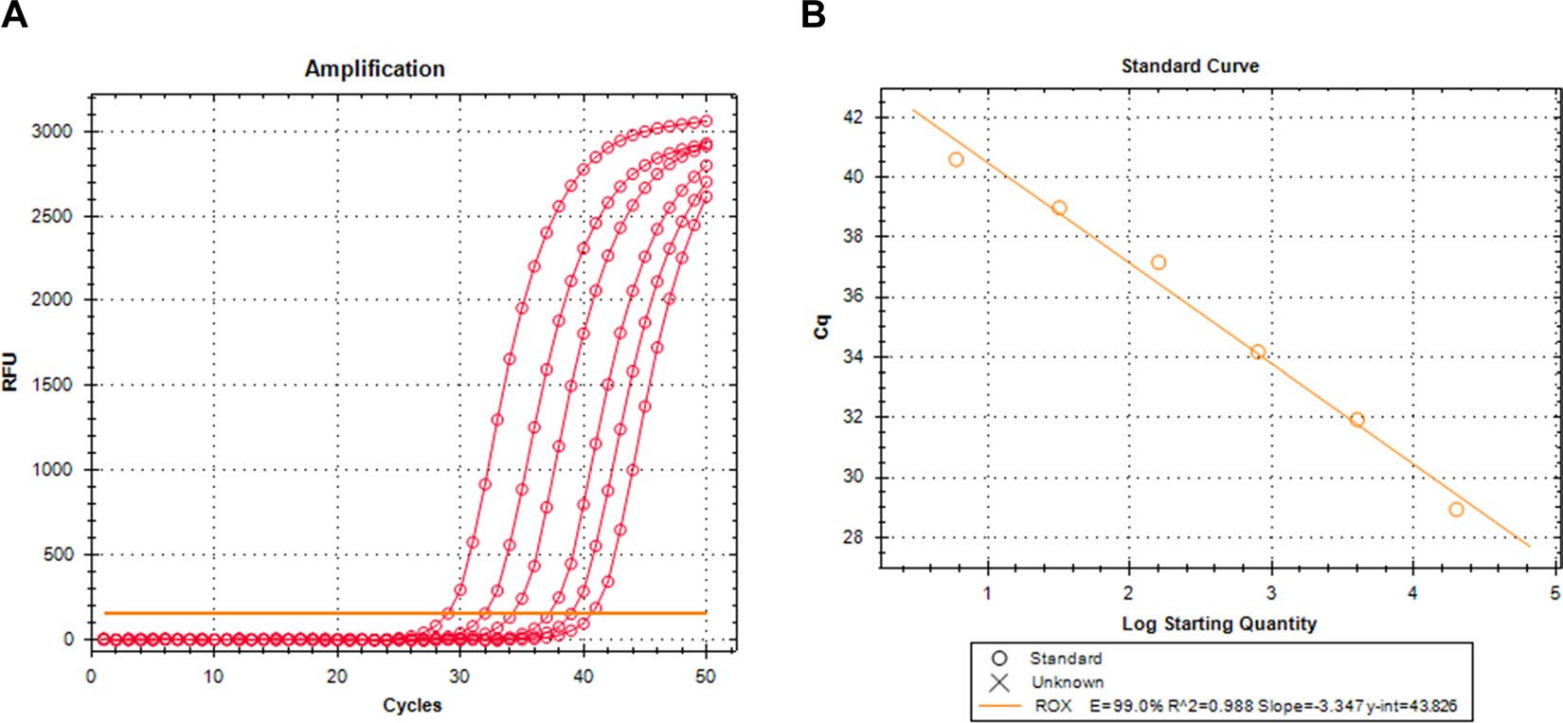
Standard curve of PCR amplification with combined primers. Real time PCR amplification was carried out with combined primers and K2-ROX(25)s probe using serially diluted wild-type DNA as template. (**A**) Amplification curves; (**B**) standard curve (*E* = 99%, *R*^2^ = 0.988).

The combined primers appear to be quite effective in real time PCR (Fig. 6), although Cq values in this case are about 5 cycles higher than those with the standard primers.

**Figure 7:**
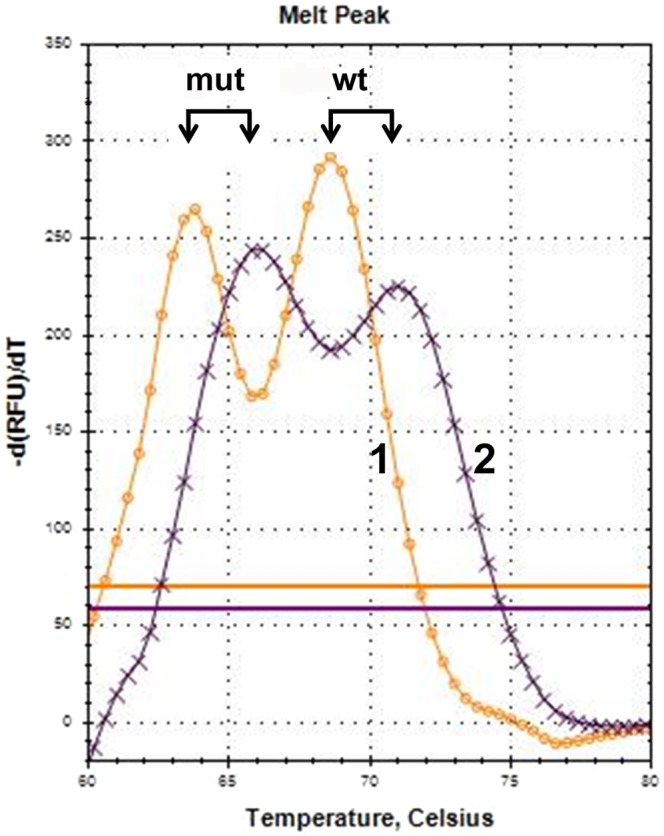
Bi-strand mutation scanning. SLAM-MS assay of *KRAS* GGC13GAC mutation was carried out with K2-ROX(25)s (curve 1) and K2-Cy5(25)as (curve 2) TaqMan probes. Positions of wild-type and mutant melt peaks are marked by arrows.

The presence in the post-PCR mixture of both sense and antisense stem-loop amplicons allows the use of either one or both TaqMan probes for melting analysis. Thus, a mutation can be scanned in any one or both amplicon strands. The option of bi-strand scanning seems preferable, because, first, it increases the reliability of the analysis (two tests differing in resolution of normal and mutant alleles complement each other), and, second, the combination of two TaqMan probes provides maximal mutant enrichment during PCR amplification (see below). An illustrative example of bi-strand SLAM-MS of a DNA sample from colorectal cancer with the *KRAS* GGC13GAC mutation shows a high resolution of wild-type and mutant melt peaks upon examination of both strands (Fig. 7). It appeared important to select the optimal sizes of the loop and probe (114 and 23–25 nucleotides, respectively, in this case). Although a detailed study of this phenomenon was not the aim of this work, we found in preliminary experiments that either increasing or decreasing the loop size diminished the DMA signal, probably due to steric hindrances of the probe fluorophore and/or quencher. It is noteworthy that specificity of the fluorophore also influences the probe hybridization ability as demonstrated by the band-shift experiments (Fig. 5).

The DMA assay permits detection of ~5% of mutant alleles in wild-type DNA^9^. A range of techniques are used for mutant enrichment during PCR. It seems likely, however, that the most economical and simplest of these is based on the fact that oligonucleotides, such as snapback primers^19^ or TaqMan probes^10^, hybridized with the template and located on the Taq polymerase pathway, reduce its extension rate^20^. Since this effect is more pronounced in the case of perfect hybrids, compared to mismatched ones, the TaqMan probes can be used for mutant enrichment. If relatively minor variances in the stability of the probe/template complexes do not give a noticeable effect under standard PCR conditions, then under suboptimal PCR conditions (for example, short PCR cycles), a strong effect of mutant enrichment and many-fold increase in sensitivity can be achieved^9,10,19^.

**Figure 8:**
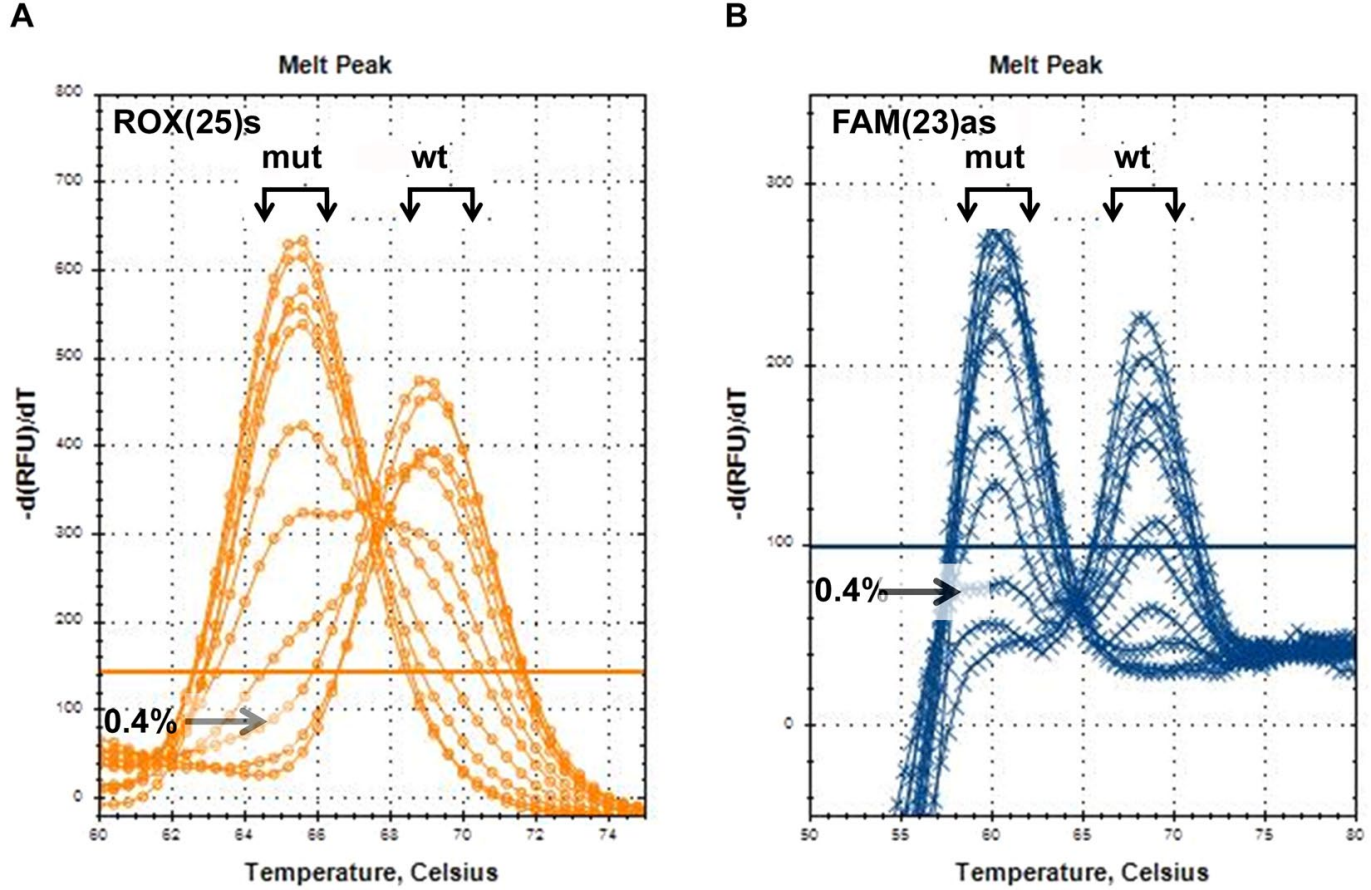
Sensitivity testing of mutant-enriched SLAM-MS assay. Sensitivity testing was carried out using SW480 DNA serially diluted with wild-type DNA. DNA samples with concentration of the mutant allele from 0% to 100% were analysed using ROX(25)s (**A**) and FAM(23)as (**B**) TaqMan probes (for convenience, the melting peaks obtained using these probes are presented separately). The wild-type and mutant melt peaks and the melt peaks of DNA samples with 0.4% mutant allele are marked by arrows.

Mutant-enriched SLAM-MS uses TaqMan probes as blocking agents in such a way that, first, 50 short PCR cycles precede 35 ordinary cycles, and, second, both, sense and antisense, TaqMan probes are present in the incubation mixture. In common, they produce strong mutant enrichment (Fig. 8). Analysis of serial dilutions of SW480 DNA in wild-type DNA demonstrates an ~10-fold increase in sensitivity of the mutant-enriched SLAM-MS (mutant allele detection limit ~0.4%) compared to the standard protocol (~5%).

**Figure 9:**
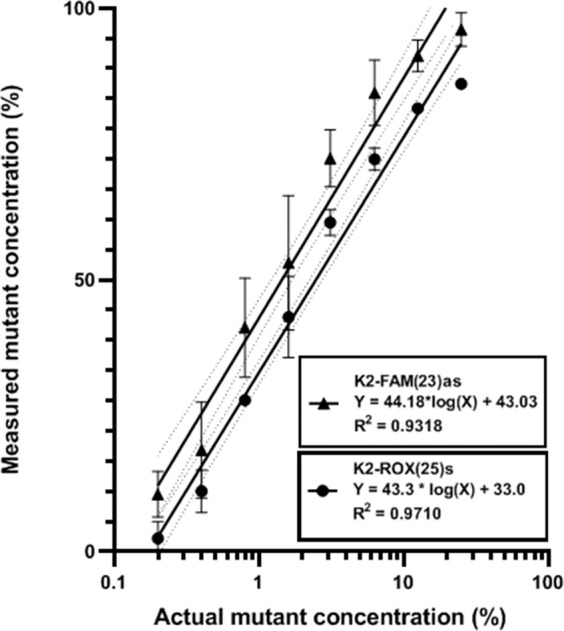
SLAM-MS quantitation. Calibration curves of mutant-enriched SLAM-MS quantitation with ROX(25)s and FAM(23)as probes were obtained within 95% confidence interval to correlate the mutant fraction measured by the PeakFit software to the actual mutant fraction (in %). Triplicate experiments were analysed; error bars indicate standard deviation. Note the logarithmic X scale. The percentage of actual mutant fraction in DNA samples can be estimated using the calibration curves.

The mutant enrichment was quantified by the signal processing procedure (see Materials and Methods) to allow determination of the mutant/wild-type allele ratios in DNA samples. A highly increased measured mutation percentage was obtained using the mutant-enriched SLAM-MS compared with the actual mutation percentage (Fig. 9). The detection limit of mutant alleles is ~0.4%.

**Figure 10:**
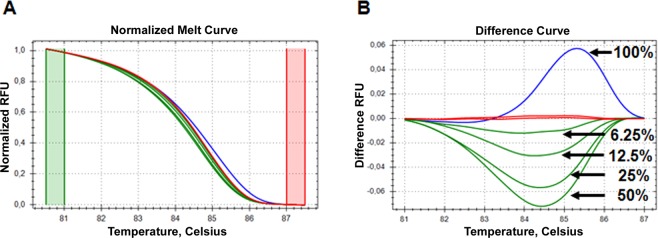
Sensitivity testing of HRM assay. Sensitivity testing was carried out using SW480 DNA serially diluted with wild-type DNA. DNA samples with concentration of the mutant allele from 0% to 100% were analysed. (**A**) Normalised melt curves. (**B**) Difference curves. The HRM assay curves (100%, 50%, 25%, 12.5%, and 6.25% mutant) are marked by arrows, wild-type curves are shown in red.

It is widely adopted that HRM (High Resolution Melting) assay is one of the most effective methods for scanning single nucleotide polymorphisms, aberrantly methylated sequences, and mutations^3,21–34^. This “closed format” method is notable for its simplicity, cost-efficiency, and sensitivity. Since it is routinely used, in particular, for the analysis of *KRAS* oncogene^3,32,34–38^, it seemed reasonable to compare its sensitivity with that of mutant-enriched SLAM-MS. Figure 10 shows the sensitivity testing of HRM assay using standard K2(114) primers and SW480 DNA serially diluted with wild-type DNA. DNA samples with concentration of the mutant allele from 0% to 100% were analysed. The detection limit of mutant *KRAS* by this method is ~6%, it is in good agreement with the literature data^35–37,39^. Thus, the mutant-enriched SLAM-MS is about an order of magnitude more sensitive than the HRM assay.

### Analysis of clinical DNA samples

**Figure 11:**
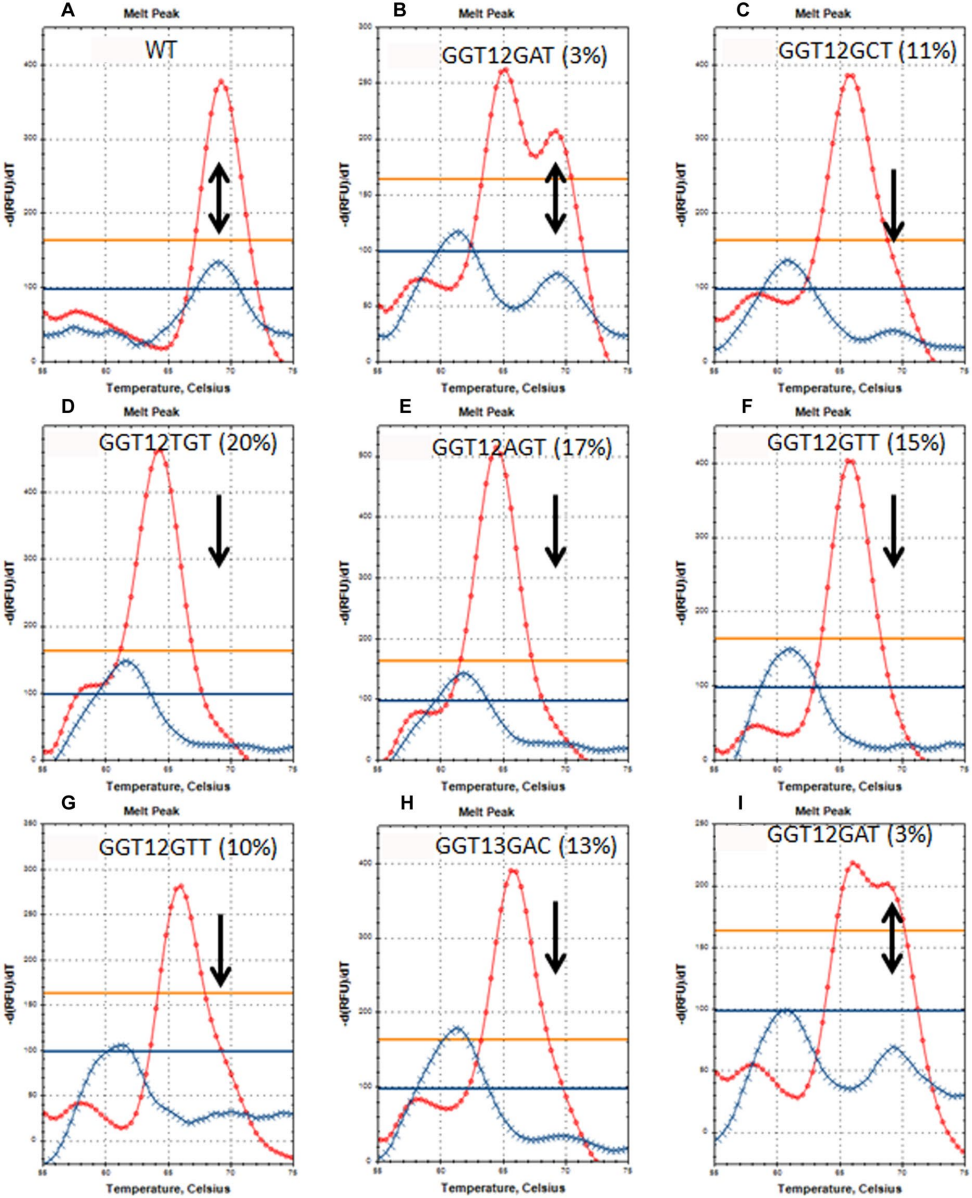
Mutant-enriched SLAM-MS of clinical DNA samples. Mutation scanning of FFPE DNA samples from normal (**A**) and colorectal cancer (**B–I**) tissues was carried out with the TaqMan probes ROX(25)s and FAM(23)as (shown in red and blue, respectively). Positions of the wild-type (WT) allele are shown by arrows. The mutation type was determined by Sanger sequencing. The actual mutant allele percentage was determined with the calibration curves presented in Fig. 9.

We used mutant-enriched SLAM-MS assay for the analysis of a panel of FFPE colorectal cancer specimens. In many of these samples, the presence of mutant *KRAS* was not detected by the standard PCR-DMA owing to its low concentration. Following enrichment, the presence of mutant *KRAS* became obvious in all samples studied and the type of each mutation was revealed by Sanger sequencing (Fig. 11). Thus, the bi-strand mutant-enriched SLAM mutation scanning allows reliable identification of the most common *KRAS* mutations.

## Discussion

To increase sensitivity of mutant detection, especially in samples like liquid biopsies, various techniques are used, such as sophisticated sequencing methods^40–42^, droplet digital PCR^43^, allele-specific PCR^7^, SNPase-AMRS qPCR^44^, COLD-PCR (co-amplification at lower denaturation temperature-PCR)^45^, HRM assay^30,32,34,46,47^, competitive probe blocking^48^, peptide nucleic acid-mediated PCR clamping^49^, and DNA terminal structure-mediated enzymatic reaction^50^. However, none of these contain all the optimal qualities: high sensitivity and specificity, simplicity and cost-efficiency, feasibility in clinical settings. Some of these techniques are expensive, others are complex and time-consuming, and many involve several successive stages and the “open tube” format which is objectionable in clinics.

The existence of different methods can be justified by the fact that DNA samples isolated from FFPE tumour tissues or blood plasma are highly variable in terms of the quantity, quality, and the percentage of mutant alleles. Since the mutant allele percentage in plasma cell-free DNA (cfDNA) varies in cancer patients as widely as from 0.1% to 60%^7,51,52^, certain methods may be warranted in specific “niches”, thus avoiding an impractical “overkill” approach. Given that the median percentage of mutant alleles in cfDNA is ~1% in patients with diverse (gastrointestinal, brain, lung, breast, head, and neck) cancers^52^, one can suggest that in the majority of cases, mutant detection is feasible using methods with a moderate sensitivity (0.1–1%). It is also noteworthy that the practically limited availability of DNA from FFPE tumour tissues and blood plasma puts a physical limit on the capabilities of ultrasensitive methods, such as ddPCR (its proclaimed detection of 0.001%, or 1 mutant in 100,000 wild-type alleles, would require 1 μg/well DNA, which is hardly feasible in practice).

Therefore, it seems reasonable to suggest a framework in which a clinical DNA sample is initially tested with the “first line” diagnostics (i.e. a simple and rapid method), and only those samples that were found negative or questionable at the initial stage and contained sufficient amount of DNA for ultrasensitive analyses, would be liable to the sophisticated and expensive “second line” diagnostics. In a clinical setting, such an approach could ensure the efficient use of time, labour, and money.

In this “proof of principle” study we showed that use of stem-loop amplicons permits rapid, simple, and quantitative bi-strand mutation scanning with a sufficiently high analytical sensitivity. The TaqMan probes widely used in genetic research make the current approach flexible and easily adaptable to a wide range of targets. In a small panel of FFPE colorectal cancer specimens, the mutant-enriched SLAM-MS showed reliable identification of the most common *KRAS* mutations confirmed by Sanger sequencing. Based on the analytical sensitivity of SLAM-MS (0.4% mutant alleles in an excess of wild-type DNA), the required DNA input would be ~3 ng. However, DNA isolated from FFPE tissues or blood plasma is extremely fragmented. As a result, the number of effective templates (determined by the size of the amplicon tested) may be significantly smaller than those suggested by spectro- or fluorimetry. Therefore, the value of 3 ng should be considered minimal. With regard to the clinical indicators of sensitivity and specificity of this method, i.e. the percentage of patients and healthy individuals identified correctly by the assay, further evaluation is needed. One can suggest that owing to the higher analytical sensitivity of the SLAM-MS over the widely used HRM assay, its diagnostic performance would be significantly higher.

Overall, the bi-strand mutant-enriched SLAM-MS is very promising as a simple, moderately sensitive, cost-effective, easy-to-use, and quantitative method of mutation scanning. It is implemented in the “closed tube format”, minimizing the risk of sample cross-contamination. In this study, it was applied for scanning mutant *KRAS*, but apparently, could be adapted for analysis of many other genes.

## Supplementary information

Supplementary Information.

## Data Availability

All relevant data are within the manuscript and its Supplementary Fig. S1 file.
