# Supplementary material for: SLAM-MS: Mutation scanning of stem-loop amplicons with TaqMan probes by quantitative DNA melting analysis
Source: Sci Rep. 2020 Mar 25;10:5476. doi: 10.1038/s41598-020-62173-x (PMC7096437; doi:10.1038/s41598-020-62173-x)
Supplement: Supplementary file 1 — Supplementary Information. [file 41598_2020_62173_MOESM1_ESM.pdf]

SLAM-MS: Mutation scanning of stem-loop amplicons with TaqMan probes by quantitative DNA melting analysis

V.N. Kondratova, I.V. Botezatu, V.P. Shelepov, & A.V. Lichtenstein*

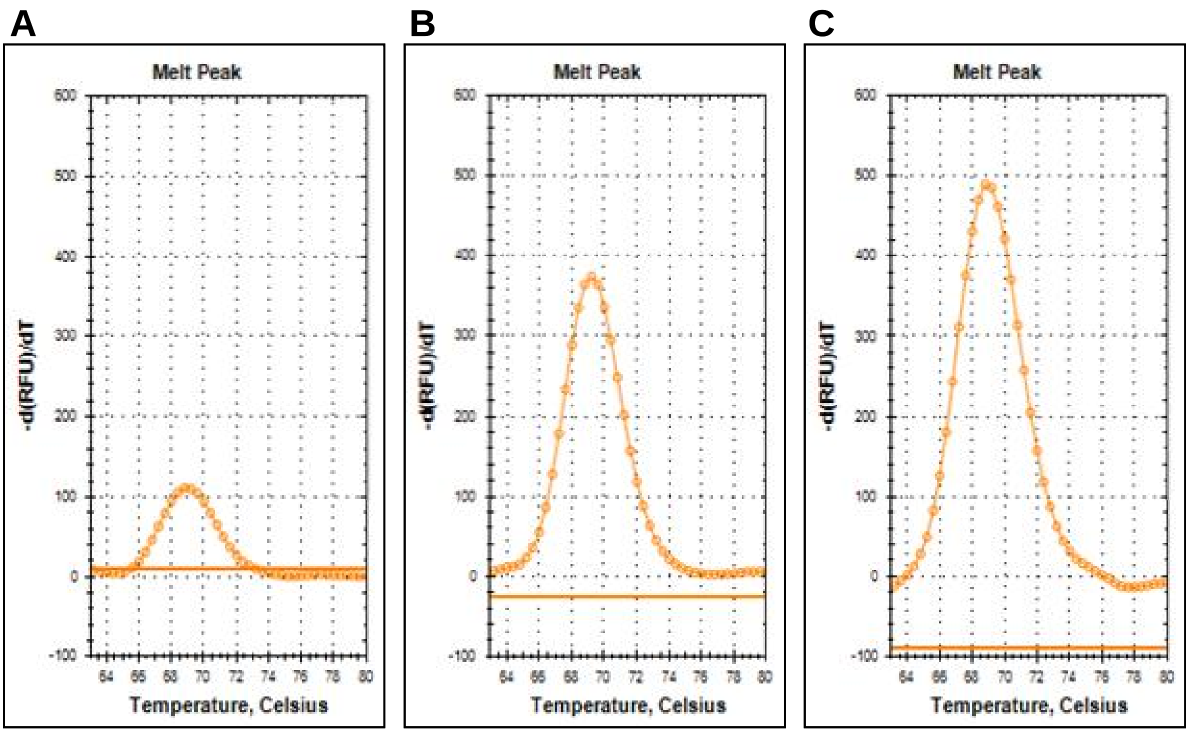

Supplementary Figure S1. Effect of renaturation cooling rate on melt peaks. A – slow cooling (at room temperature); B - cooling directly in the instrument; C - quick cooling (by immersing the plate in a water–ethanol mixture pre-cooled to -20 °C).
